# 

**Published:** 1893-09

**Authors:** 


					﻿Vol XIII.
SEPTEMBER, 1893.
NO. 9>
THE
pOMEDPATfflC pHY£lClA]\l,
A Monthly Journal of Homoeopathic Materia Medica
and Clinical Medicine.
"He it a freeman whom truth makes free, and all are slaves beside."
"Seek the Truth: come whence it may, cost what it will."
XDITED AND PUBLISH KD BY
WALTER NT. JAMES, NT. D.
PHILADELPHIA :
No. 1125 spruce street.
LONDON:
ALFRED HEATH & CO., Solk Agbnts tor Obkat Britain,
114 Ebury Street, Eaton Square.
Yearly Subscription, $2.50, invariably in advance. Single numbers, 96 ets.
Entered at the Philadelphia PeeVOffiee M Beoood*Claes Mall Matter.
				

